# Reviewer Acknowledgement 2014

**DOI:** 10.1186/s13047-015-0060-2

**Published:** 2015-03-28

**Authors:** Hylton B Menz, Mike Potter

**Affiliations:** La Trobe University, Melbourne, Australia; University of Southampton, Southampton, United Kingdom

## Abstract

The Editors of *Journal of Foot and Ankle Research* would like to thank all our reviewers who have contributed to the journal in Volume 7 (2014).

**Pippa Anderson**

United Kingdom

**John Arnold**

Australia

**Stuart Baird**

United Kingdom

**Christian John Barton**

Australia

**Heiner Baur**

Switzerland

**Paul Bennett**

Australia

**Shan Bergin**

Australia

**Adam Bird**

Australia

**Chris Bishop**

Australia

**Daniel Bonanno**

Australia

**Katherine Burgess**

United Kingdom

**Joshua Burns**

Australia

**John Burston**

United Kingdom

**Paul Butterworth**

Australia

**Matthew Carroll**

New Zealand

**Alex Chamberlain**

Australia

**Graham Chapman**

United Kingdom

**Angus Chard**

Australia

**Nicole Chimera**

USA

**Lisa Chinn**

USA

**Vivienne Chuter**

Australia

**Mark Cornwall**

USA

**Matthew Cotchett**

Australia

**Emma Cowley**

United Kingdom

**Sarah Curran**

United Kingdom

**Hamish Curry**

Australia

**Annette Davis**

Australia

**Sophie De Mits**

Belgium

**Sharon Dixon**

United Kingdom

**Stephen Duckett**

Canada

**Angela Evans**

Australia

**Lisa Farndon**

United Kingdom

**Gwen Fernandes**

United Kingdom

**Jill Ferrari**

United Kingdom

**Joseph Fiorito**

USA

**Alex Forrester**

United Kingdom

**Jacqueline Forss**

United Kingdom

**Andrew Franklyn-Miller**

Australia

**Nicoletta Frescos**

Australia

**Tetsuo Fukunaga**

Japan

**Alfred Gatt**

Malta

**Kathleen Gatt**

Malta

**Moshi Geso**

Australia

**Paul Gibbons**

Australia

**Uri Givon**

Israel

**Kelly Gray**

Australia

**Joel Gurr**

Australia

**David Gutekunst**

USA

**Jill Halstead**

United Kingdom

**Joseph Hamill**

USA

**Farina Hashmi**

United Kingdom

**Fiona Hawke**

Australia

**Gordon Hendry**

United Kingdom

**Claire Hiller**

Australia

**Hiroaki Hobara**

Japan

**Sheree Hurn**

Australia

**Kasper Janssen**

The Netherlands

**Robert Kidd**

Australia

**Tim Kilmartin**

Ireland

**Kevin Kirby**

USA

**Dorit Kunkel**

United Kingdom

**Toshiyuki Kurihara**

Japan

**Peter Lazzarini**

Australia

**Alberto Leardini**

Italy

**Szu-Ping Lee**

USA

**Pazit Levinger**

Australia

**Rod Ling**

Australia

**Anmin Liu**

United Kingdom

**Anna Lorimer**

New Zealand

**Jody Lucas**

United Kingdom

**John Ludlow**

USA

**Claire MacGilchrist**

Ireland

**Nicola Maffulli**

United Kingdom

**Ryan Mahaffey**

United Kingdom

**Nele Mahieu**

Belgium

**Matthew Malone**

Australia

**Ian Mathieson**

United Kingdom

**Julie McDonald**

Australia

**Andrew McMillan**

Australia

**Karen Mickle**

Australia

**Stewart Morrison**

United Kingdom

**George S Murley**

Australia

**Vanessa Nube**

Australia

**Susan Pager**

Australia

**Kade Paterson**

Australia

**Craig Payne**

Australia

**Byron Perrin**

Australia

**Carlos Pique-Vidal**

Spain

**Cameron Powden**

USA

**Trevor D Prior**

United Kingdom

**Smita Rao**

USA

**Anita Raspovic**

Australia

**Michael Skovdal Rathleff**

Denmark

**Anthony Redmond**

United Kingdom

**Edward Roddy**

United Kingdom

**Keith Rome**

New Zealand

**Thomas Roukis**

USA

**Hagen Schmal**

Germany

**Debbie Sharman**

United Kingdom

**Zhongmin Shi**

China

**Sorin Siegler**

USA

**Elizabeth Skinner**

Australia

**Simon Smith**

Australia

**Jinsup Song**

USA

**Martin Spink**

Australia

**Julie Stebbins**

United Kingdom

**Martijn Steultjens**

United Kingdom

**Robert Stoekenbroek**

The Netherlands

**Geoff Suissman**

Australia

**Luke Taylor**

Australia

**Scott Telfer**

United Kingdom

**Dominic Thewlis**

Australia

**Kirsten Tulchin-Francis**

USA

**Stephen Urry**

Australia

**Babette Corine van der Zwaard**

The Netherlands

**Tom van Raaij**

The Netherlands

**Wesley Vernon**

United Kingdom

**Scott Wearing**

Australia

**Anita Williams**

United Kingdom

**Sebastian Wolf**

Germany

**David Wylie**

United Kingdom

**Yayi Xia**

China

**Maria Young**

United Kingdom

**Bernhard Zipfel**

South Africa

